# The Dangers Arising from Syphilis in the Practice of Dentistry

**Published:** 1891-02

**Authors:** L. Duncan Bulkley

**Affiliations:** Attending Physician to the New York Skin and Cancer Hospital, etc.


ARTICLE VII.

### THE DANGERS ARISING FROM SYPHILIS IN THE PRACTICE OF DENTISTRY.

BY L. DUNCAN BULKLEY, A. M., M. D.

[Attending Physician to the New York Skin and Cancer Hospital, etc.]

We will now consider some of the observed facts in regard to the communication of syphilis in dentistry, and afterwards examine the modes of transmission and the means of prevention. Our clinical study will naturally divide itself into two lines of thought: 1, in regard to the dangers from syphilis to *patients* undergoing dental operations; and 2, in regard to dangers to the *operator* from the same source.

First as to the dangers to the *patient* from exposure to the syphilitic poison during dental operations:

Inasmuch as it presents many points of interest, relating both to the patient and operator, I may be allowed first to recite the case alluded to, which came under my own observation and treatment, and which first called my attention particularly to the subject.

Mr. X. W., a gentleman of intelligence and position, aged 60 years, came to me September 11, 1884, on account of a sore on the tongue, which he feared to be a cancer. The history was, that some ten weeks before his first visit, he had first noticed a little point of soreness, which had gradually increased in size, in spite of treatment, until latterly it had come to give him considerable annoyance, so that he was conscious of its presence at all times; the true nature of the sore had evidently not been recognized.

On examination, there was found on the right side of the tongue, about an inch from its tip, a hard, inflamed mass, nearly half an inch in diameter, the center ulcerating and the edges somewhat everted; it was not painful except when irritating food or drink touched it. The two upper molars were found to have sharp and rough edges, and he had been wearing a red rubber plate until recently. There was a small and painful gland beneath the jaw on that side, slightly enlarged.

Thinking that the ulcer might possibly be due to irritating local causes, he was given a soothing mouth wash, and an alkali internally. Five days later there was a marked improvement in its condition; the ulcer had a less angry look, but its edge was more clearly defined as the inflammatory element had somewhat subsided. He had been, of his own accord, to his regular dentist, and had had the roughened teeth made smooth, and had left out his set of artificial teeth.

From a careful second study of the case, I then felt convinced that the sore was a chancre, a primary lesion of syphilis, and he was immediately put on antisyphilitic treatment; the general eruption and other symptoms which followed a few weeks later rendered the diagnosis certain, together with the remarkable manner in which the sore healed and symptoms vanished under the proper treatment for syphilis.

In searching for the mode by which the syphilitic poison had gained entrance, it was learned that, during the month or so previous to the appearance of the sore upon the tongue, he had, through the persuasion of a friend, been under the care of a dentist of the cheaper, advertising order, who, he had noticed, was not at all cleanly either in his person or with his instruments. He could not locate the exact date of the injury of the tongue by the dental instruments, but work had been done in that locality, and he remembered the instrument occasionally slipping, as will often happen, inflicting injury to the soft parts. He was a married man with a family, and was very desirous of learning how he had become infected; he had certainly not been exposed in sexual intercourse, nor in any other manner which we could discover.

The interesting points in the case are: First, that while the proof is not absolute that he was infected in the dentist's chair, still the circumstantial evidence is so strong that little, if any, doubt can be entertained that the poison came through this channel. The habits and ways of the particular dentist were such that poisonous material from the mouth of a previous syphilitic patient could readily have been transferred on instruments, or otherwise, to the wound made in the tongue, either by the sharp teeth or by a slip of an instrument. The second interesting point is, that this patient, before the true nature of the disease was ascertained, had been to his own regular dentist for smoothing the teeth, and so had certainly exposed him, and others through him, to the poison, which was secreted freely from the raw surface of the chancre.

The earliest recorded cases of the transmission of syphilis in dental operations are in connection with the transplantation of teeth, during the last quarter of the eighteenth century.

Sir William Watson * published a case of this descrip-

---

* Watson, "Transactions of College Surgeons," 1785. iii., p. 328.

tion, and John Hunter † relates two similar cases about which there can be no doubt. J. C. Lettsom ‡ also recorded three cases; of these, one was personal, one seen by a Dr. Hamilton, and the third occurred in America, having been observed by Kuhn, in Philadelphia; these gentlemen furnished notes of the cases to Dr. Lettsom. This mode of transmission does not occur again in literature, to the knowledge of the writer, although Gibier § says that "Cases have been recently related." In view, however, of a recent revival of the operation of tooth transplantation, or implantation, it is quite possible that the future may furnish fresh instances of this mode of the innocent acquiring of syphilis.

From this period no other causes of the transmission of syphilis through dental procedures are found recorded for nearly a century; indeed, not until the advent of modern operative dentistry and active medical observation.

The first case met with is one reported by Dr. C. W. Dulles, ‖ of Philadelphia, and which was also seen by the late Dr. Maury. The patient, a female domestic of excellent character, developed a chancre of the lip two weeks after a visit to a dentist; on that occasion he extracted a tooth, and later did some cleansing of the teeth. Although no confirmation was obtained, it seemed reasonable to suppose that the operation of extraction was in some way responsible for the inoculation.

Dr. F. N. Otis * also mentions a chancre of the lip which occurred in a gentleman "about three weeks after a morning spent in a dentist's chair."

Lancereaux † relates a similar case of chancre of the lower lip in a woman, after the extraction of a tooth and

† Hunter, "Treatise on the Venereal Disease," 1st. Engl ed., 1786: 1st Amer. ed., Philadelphia, 1818, p. 362.

‡ Lettsom, "Transactions Lon. Med. Soc.," vol. i., 1787, p. 137.

§ Gibier, "Ann. de Dermat. et de Syph.," 1882, p. 129.

‖ Dulles, "Phila. Med. and Surg. Reporter;" Jan., 1878.

* Otis, "Lecture on Syphilis," New York, 1881, p. 102.

† Lancereaux, "Proc. Acad. de Med. de Paris," "Union Med.," 1889, xlviii., p. 655.

other dental work, and Giovannini,‡ of Bologna, has reported a chancre of the lip, apparently from a dentist's instrument.

Leloir § mentions having seen a man with chancre of the gum, in whom the infection seemed to have taken place in consequence of cleaning and filling a cavity in a tooth, with soiled instruments. Lydston ‖ has likewise reported the case of a woman with syphilis, in whom the chancre on the gum, below the lower middle incisors, appeared to be the result of some cleaning and repairing of the teeth done three weeks previously; the glands beneath the jaw were enlarged, beginning a week or more after the appearance of the sore on the gum.—*Extract from Paper read before the New York Odontological Society, April, 1890, and printed in the International Dental Journal.*

---

ARTICLE VIII.

### SIMPLE METHOD FOR CONTROLLING EPISTAXIS.

‡ Giovannini, "Le Sperimentale," 1889, p. 262.

§ Leloir, "Lecon sur la Syphilis," 1886, p. 62.

‖ Lydston, "Jour. Amer. Med. Assoc., 1886, vi., p. 654.

